# Identifying potential drugs for treating Cardiovascular-kidney metabolic syndrome via reverse network pharmacology

**DOI:** 10.3389/fphar.2025.1627236

**Published:** 2025-06-30

**Authors:** Si’ao Wen, Ao’ni Fu, Fen Liu, Yayu You, Linkai Li, Wen Xiao, Haoran Zhong, Xiuqin Hong, Xin Zhong, Yongjun Hu, Zhengyu Liu

**Affiliations:** ^1^ Department of Cardiology, Hunan Provincial People’s Hospital/The First Affiliated Hospital of Hunan Normal University, Changsha, China; ^2^ Institute of Cardiovascular Epidemiology, Hunan Provincial People’s Hospital, Changsha, Hunan, China; ^3^ Institute of Epidemiology, Hunan Provincial People’s Hospital, Changsha, Hunan, China; ^4^ Clinical Research Center for Heart Failure in Hunan Province, Changsha, Hunan, China; ^5^ Leading Unit of Cardiovascular Prevention and Treatment Technology of Hunan Province, National Health Commission, Changsha, Hunan, China; ^6^ Key Laboratory for Arteriosclerology of Hunan Province, Hunan International Scientific and Technological Cooperation Base of Arteriosclerotic Disease, Hengyang Medical School, Institute of Cardiovascular Disease, University of South China, Hengyang, Hunan, China; ^7^ Department of Ultrasonic Medicine, Hunan Provincial People’s Hospital (The First Affiliated Hospital of Hunan Normal University), Changsha, China

**Keywords:** Cardiovascular-kidney metabolic syndrome (CKM), Berberine, network pharmacology, “two-hit” model, animal model, HFpEF

## Abstract

**Background:**

Cardiovascular, Kidney and metabolic syndrome (CKM) is a complex disease, for which current therapeutic approaches have limited efficacy. This study aims to screen for potential targets and novel drugs for treating CKM using network pharmacology.

**Methods:**

Using reverse network pharmacology, core targets and potential drugs for CKM were identified. Candidate compounds were screened from a natural product library. Male C57BL/6J mice were fed a high-fat L-NAME diet for 12 weeks to induce CKM and confirm successful model establishment, followed by 4 weeks of BBR (Berberine) treatment. Metabolic parameters, as well as cardiac and renal structural and functional indices, were assessed. Key targets and potential drugs identified through network pharmacology and bioinformatics were validated using pathological analysis, RT-qPCR, and Western blotting (WB), collectively demonstrating the therapeutic effects of BBR on CKM.

**Results:**

Network pharmacology identified multiple core targets of CKM, and reverse pharmacology discovered the potential drug BBR (Berberine) from a natural product library. *In vivo* experiments demonstrated that the “two-hit” HFpEF model, which is induced by a high-fat diet combined with L-NAME treatment for 12 weeks and is characterized by metabolic disorders, cardiac diastolic dysfunction, and renal fibrosis, can be used as a new model of CKM. BBR improved metabolic disorders, cardiac diastolic function, and renal damage in CKM mice by regulating lipid metabolism, glucose metabolism, and fibrosis-related pathways.

**Conclusion:**

The “two-hit” HFPEF model can be used as a new model of CKM, and BBR may become a new candidate drug for the treatment of CKM through multiple targets.

## 1 Introduction

Obesity, diabetes, and CKD are highly prevalent, often occurring simultaneously, and significantly increase the incidence and mortality of cardiovascular disease. The mechanisms of these four disease states are also closely intertwined, with multidirectional relationships, common risk factors, and shared therapeutic targets. Given the complex interactions between these diseases, the American Heart Association (AHA) recently proposed a new comprehensive health disorder known as the Cardiovascular-Kidney-Metabolic Syndrome (CKM) (Ndumele et al., 2023). This syndrome expands on the traditional concept of cardio-renal syndrome (Cardiorenal Syndrome, CRS), emphasizing the central role of metabolic risk factors in the interplay between heart and kidney damage (Savira et al., 2020; Young and Eknoyan, 2024) In recent years, the incidence of CKM Syndrome has significantly increased, becoming one of the major diseases threatening public health (Young and Eknoyan, 2024). The syndrome is characterized by the coexistence of multiple diseases that interact, exacerbate conditions, and increase treatment complexity (Savira et al., 2020). It has a close association with other diseases such as coronary artery atherosclerosis, chronic kidney disease (CKD), obesity, hypertension, and hyperlipidemia (Cosentino et al., 2023). These diseases have shared pathogenic mechanism, and research on these shared pathways is crucial for understanding the pathogenesis of CKM Syndrome and identifying effective treatment strategies (Cosentino et al., 2023). For the current treatment of CKM, renin-angiotensin system inhibitors and sodium-glucose cotransporter 2 inhibitors can slow kidney disease progression in most CKD patients and reduce heart failure complications. Nonsteroidal mineralocorticoid receptor antagonists like finerenone and glucagon-like peptide-1 receptor agonists like semaglutide also provide cardiorenal benefits, with semaglutide effectively reducing body weight (Zhu et al., 2025). However, there is insufficient evidence supporting efficacy for the CKM syndrome spectrum (such as early metabolic abnormalities) (Cortés-Camacho et al., 2024; Vaduganathan et al., 2024), so it is urgent to develop cheap, accessible and common drugs for CKM.

Due to the complex etiology of Cardiovascular-kidney metabolic syndrome, which involves multiple organs including the heart, kidneys, adipose tissue, and liver, there is currently no animal model that can fully simulate this syndrome. The current model studies of CKM mice exhibit the following major shortcomings. Most models only simulate single metabolic abnormalities (such as hypertension or obesity) (Kennedy et al., 2010; Dragoljevic et al., 2021), making it difficult to fully replicate the multi-system disease characteristics of CKM. For example, models using only HFD (high-fat diet) lack stable renal impairment phenotypes, while chronic kidney disease models often lack indicators of metabolic disorders (Carvalho et al., 2024). Current models fail to adequately simulate cardiorenal interactions, such as the low co-occurrence rate of cardiac diastolic dysfunction and renal fibrosis (Valero-Muñoz et al., 2021), and lack critical pathological features like vascular endothelial dysfunction. Meanwhile, We observed the feasibility advantages of the classic HFPEF model: HFD + L-NAME “two-hit” HFPEF model (Schiattarella et al., 2019), in which L-NAME, a nitric oxide synthase (NOS) inhibitor, reduces NO production by inhibiting endothelial NOS (eNOS), leading to elevated blood pressure and hemodynamic disturbances, and is often combined with a high-fat diet (HFD) to construct HFpEF models. Additionally, its impact on renal function and structure has received significant attention. For example, L-NAME can significantly increase serum creatinine and urea levels by inhibiting NO production, indicating decreased glomerular filtration rate and tubular damage (Ragab et al., 2021). L-NAME causes interstitial fibrosis, glomerulosclerosis, and thickening of the vascular wall, which are structural changes consistent with the pathological features of chronic kidney disease (CKD) (Prince et al., 2020). It has been proven that this model shows significant responses to CKM-targeting drugs such as SGLT2 inhibitors (e.g., empagliflozin) (Levin et al., 2020; Shi et al., 2024; Herrington et al., 2025), Therefore, we utilized HFD + L-NAME model to establish CKM mice model in this study.

**FIGURE 1:**
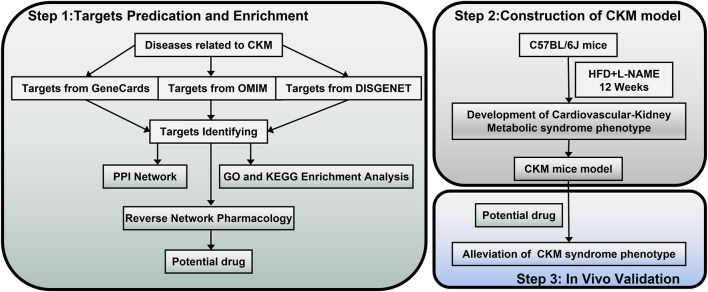
Flowchart of this stydy. Part 1: Targets prediction and enrichment. Data were collected from GeneCards, OMIM, and DisGeNET to identify targets related to CKM. Subsequently, the protein-protein interaction (PPI) network was constructed, and Gene Ontology (GO) and Kyoto Encyclopedia of Genes and Genomes (KEGG) enrichment analyses were performed to predict potential drugs through reverse network pharmacology. Part 2: Construction of the CKM model. CKM mouse model was developed using C57BL/6J mice treated with HFD and L-NAME for 12 weeks to establish a cardiovascular-kidney metabolic syndrome phenotype. Part 3: *In vivo* validation. Potential drugs were administered to the CKM mice model to validate their efficacy in alleviating the CKM syndrome phenotype.

Currently, with the rapid development of bioinformatics, network pharmacology based on large databases has become a powerful tool for detailing the mechanisms of complex drug systems from the molecular to the pathway level. Network pharmacology integrates multidisciplinary technologies such as systems biology, polypharmacology, molecular network data, bioinformatics, and computer simulations, making it highly suitable for analyzing targets and corresponding compounds or herbs involved in multiple diseases (Lee et al., 2024; Zhai et al., 2025). Many studies have used network pharmacology methods to reveal the mechanisms by which drugs affect diseases (Yang et al., 2022). This approach has become a promising method for accelerating drug research and development (Li et al., 2023). However, there is currently no systematic network pharmacology study on CKM, and reports identifying drugs through intersecting targets between clinical syndromes are also few. In this study, reverse network pharmacology was applied to analyze CKM-related gene/protein networks for potential therapeutic targets, which were then validated for efficacy and safety in animal experiments. The flowchart of this study is shown in Figure 1.

## 2 Materials and methods

### 2.1 Network pharmacology part

#### 2.1.1 Target screening and network construction

Currently, major databases lack disease entries directly related to CKM. According to the definition of CKM syndrome staging proposed by the American Heart Association Scientific Statement and Presidential Advisory (Young and Eknoyan, 2024), the first stage is characterized by excessive or dysfunctional obesity as the initiating factor. The second stage involves high-risk metabolic factors such as hyperlipidemia, hypertension, and diabetes. The third stage includes clinical diseases like atherosclerosis, coronary atherosclerotic heart disease, and chronic kidney disease (Zoccali et al., 2024). To address this gap, we searched the OMIM database (https://omim.org/), GeneCards (https://www.genecards.org/), and DisGeNET (https://www.disgenet.org/) using keywords such as “hyperlipidemia,” “diabetes,” “obesity,” “hypertension,” “atherosclerosis,” “coronary heart disease,” and “chronic kidney disease (CKD).” Relevant genes were screened, retaining target data from OMIM and DisGeNET. In the GeneCards database, targets were chosen based on the top 25% of the Relevance Score, which indicates the significance of the gene’s association with the disease. After eliminating duplicate targets for each disease, we constructed a Venn diagram using the Wei Sheng Xin platform (https://www.bioinformatics.com.cn/) to identify 183 common targets across the seven diseases.

#### 2.1.2 Protein interaction network (PPI) and pathway enrichment analysis

The 183 common targets were imported into the STRING database, and the interaction network was constructed with the highest confidence >0.9. The protein interaction network was exported and then imported into Cytoscape 3.10.1 for visualization. The cytoNCA plugin was utilized to obtain the score, while the Cyto Hubba plugin was employed to identify the top 10 genes in the PPI network and to construct a critical sub-network. Additionally, cross-gene pathway analysis was performed using the Kyoto Encyclopedia of Genes and Genomes (KEGG) pathway enrichment via the DAVID Bioinformatics Resources (https://david.ncifcrf.gov/). The 183 obtained targets were also imported into the STRING database to construct a PPI network. With the highest confidence level (0.900) selected, it resulted in a network with 169 nodes and 440 edges. This network was then imported into Cytoscape software to build the core PPI network diagram. Using the CytoNCA plugin, key targets were screened based on the relationships between edges and nodes. The degree centrality (betweenness) and closeness centrality (closeness) were obtained. Targets with degree centrality >116.4756497 (median) and closeness centrality >0.187910644 (median) were selected.

#### 2.1.3 Gene-compound-herb network

The 183 targets were imported into the BATMAN-TCM database (Kong et al., 2024) or matching, and the obtained targets, compounds and herbs were visualized in Cytoscape 3.10.1.

### 2.2 Animals

#### 2.2.1 CKM animal model construction

For mice, all the experiments were approved in advance by the Institutional Animal Care and Use Committee of Hunan Provincial People’s Hospital (First Affiliated Hospital of Hunan Normal University) (Biomedical Research Ethical Approval number: Lunli Examination Section 2022 No. 172). Eight week old male C57BL/6 mice (18–22 g) were purchased from Hunan Huan Yu Biotechnology Co., LTD. Experimental Animal Co. Ltd., Changsha, Hunan, China (animal qualification certification: No. 410983241100868114). The mice were housed in a controlled environment with temperatures maintained between 20°C and 26°C, relative humidity levels kept between 30% and −0%, and a 12-h light-dark cycle. They had unrestricted access to tap water and a standard pellet diet. The utilization of animals in research adhered to national regulations governing experimental animal use.

#### 2.2.2 “two-hit” induced CKM mouse model

Mice were fed a high-fat diet (HFD; XTHF60, Xietong Shengwu Co. Ltd., China) combined with L-NAME (L-NAME; HY-18729A, MedChemExpress Monmouth Junction, NJ, United States) (0.5 g/L in drinking water) in their drinking water for 12 weeks. The control group (Control) was fed only the HFD and provided with regular drinking water. After modeling, the success of the model was confirmed through echocardiography, serum indicators (NT-proBNP, creatinine, uric acid, elevated lipid levels), and pathological analysis (increased renal fibrosis, glomerular damage).

#### 2.2.3 Experimental grouping and drug intervention

Mice were randomly divided into 4 groups (n = 5/group): Control (Control): HFD + 0.5% sodium carboxymethyl cellulose (CMC-Na; HY-Y0703 800–1,200 mPa.s MedChemExpress Monmouth Junction, NJ, United States) gavage. Model Group (CKM + Vehicle): HFD + L-NAME + CMC-Na gavage. BBR (Berberine Hydrochloride, Yun Peng pharmaceutical Group) Low Dose Group (Low-BBR): CKM model + BBR (50 mg/kg/d) gavage. BBR High Dose Group (High-BBR): CKM model + BBR (100 mg/kg/d) gavage. The drug intervention lasted for 4 weeks. The doses and duration of berberine treatment were selected based on previous studies (Abudureyimu et al., 2023; Sun et al., 2023).

#### 2.2.4 Physiological and metabolic index detection

##### 2.2.4.1 Weight and blood pressure

Weight was recorded weekly, and systolic blood pressure (SBP) and diastolic blood pressure (DBP) were measured by tail sleeve method (Softron BP-98A).

##### 2.2.4.2 Serological indicators

After blood collection, the blood was mixed in a centrifuge tube and then centrifuged at 2000 g for 10 min at 4°C. The upper plasma was collected for further analysis. (1) Lipids: Total serum cholesterol (TC), triglycerides (TG), and low-density lipoprotein (LDL-C) were measured using an automated biochemical analyzer (BS-360S, Mindray Bio-medical Electronics Co., Ltd., Shenzhen). (2) Serum NT-proBNP: The upper plasma was taken to measure the level of NT-proBNP in serum according to the instructions of the kit (JL11641, Jianglai biology, China). (3) Creatinine (SCr), blood urea nitrogen (BUN), and uric acid (UA) were determined using a biochemical analyzer (BS-360S, Mindray Bio-medical Electronics Co., Ltd., Shenzhen). (4) Serum IL-1β, TNF-α, and IFN-γ levels were measured using ELISA kits according to the manufacturer’s instructions (IL-1β-JL18442, TNF-α-JL47783, and IFN-γ-JLW10967, Jianglai Biology, China).

##### 2.2.4.3 Glucose tolerance (GTT) and insulin resistance test (ITT)

One week prior to the experiment, the mice were acclimated and pre-stimulated by gently stroking their tails to minimize stress. Mice underwent an overnight fast prior to the determination of baseline blood glucose levels (mg/dL) using 10 μL of tail vein blood in a glucose meter (Sinocare, China). Intraperitoneal injection of glucose (2 mg dextrose/g body weight) in sterile PBS was administered, and blood glucose levels were monitored at various time points (0, 15, 30, 60, 120 min) post-injection. Insulin tolerance testing was performed using the same glucometer after a 6-h fast. After establishing baseline glucose levels, the mice was administered an intraperitoneal injection of insulin (0.75 U/kg) to evaluate insulin sensitivity. The clearance of plasma glucose was then monitored at the specified time points following the injection. GTT and ITT tests were performed 1 week apart.

##### 2.2.4.4 The heart function was assessed by ultrasound

Conventional echocardiography and Doppler imaging were performed using an animal ultrasound imaging system (VINNO 6, Beijing, China). Anesthesia was administered with isoflurane, with an initial induction at 5% followed by maintenance at 2%. Left ventricular ejection fraction and other parameters of systolic function were in review derived from short axis M-mode scans at the mid-ventricular level identified by the presence of papillary muscles in anesthetized mice. Diastolic function measurements were obtained using pulsed-wave mode and tissue Doppler under apical 4-chamber view. The following parameters were collected: heart rate (HR), left ventricular end-diastolic diameter (LVIDd), left ventricular end-systolic diameter (LVIDs), end-diastolic interventricular septal wall thickness (IVSd), left ventricular end-diastolic posterior wall (LVPWd), left ventricular fractional shortening (LVFS), left ventricular ejection fraction (LVEF), peak Doppler blood inflow velocity across the mitral valve during early diastole (E), peak Doppler blood inflow velocity across the mitral valve during late diastole (A), and peak tissue Doppler of myocardial relaxation velocity at the mitral valve annulus during early diastole (E’). Following the completion of the procedures, all mice successfully emerged from anesthesia without any complications. Each parameter was measured a minimum of three times, and the resulting averages are provided.

#### 2.2.5 Pathological analysis

The harvested hearts, livers and kidneys were fixed in 4% buffered formaldehyde for 24 h, embedded in paraffin, and sectioned into 5 µm thick slices. Hematoxylin-eosin staining (H&E staining) and Masson’s trichrome staining were conducted.

Heart and kidney sections were examined under a microscope to identify collagen deposition, which appeared as blue staining indicative of fibrosis. The quantification of collagen in the heart and kidney sections was performed using ImageJ software (https://imagej.net/software/fiji/downloads), with the average area measured across ten image fields to assess interstitial fibrosis (proportion of collagen fiber area (%) = collagen pixel area/visual field pixel area ×100%).

The glomerular sclerosis score was calculated as follows: 0 for normal glomeruli; 1 point for mesangial expansion or sclerosis area <25%; 2 points for 25%–50% sclerosis area; and 3 points for 50%–75% sclerosis area. A total of 20 glomeruli from each specimen were examined under a light microscope at ×400 magnification, and the average value was used as the glomerular sclerosis index. The tubulointerstitial score was calculated based on the extent of lesions (tubular atrophy, cast formation, interstitial inflammation, and fibrosis) as follows: 0 points for no tubulointerstitial damage; 1 point for lesion range <25%; 2 points for lesion range 25%–50%; and 3 points for lesion range >50%. Ten fields from each specimen were examined under a light microscope at ×100 magnification, and the average value was used as the renal interstitial tubular lesion index.

The wheat germ agglutinin staining (WGA staining) was used to label the cell membranes of cardiomyocytes. The cross-sectional areas of cardiomyocytes in the left ventricle (LV) were visualized using WGA staining at a concentration of 5 μg/mL (Servicebio, China) and quantified by measuring single myocyte cross-sectional areas with ImageJ software (https://imagej.net/software/fiji/download). The cardiomyocyte cell size area (measured in mm^2^) was assessed by analyzing 10 images per section.

#### 2.2.6 Real-Time quantitative polymerase chain reaction analysis

Total RNA was extracted from tissues using Trizol reagent (Invitrogen, Shanghai, China) according to the manufacturer’s instructions. First-strand cDNA was synthesized using the RevertAid First Strand cDNA Synthesis Kit (Thermo Fisher Scientific, Shanghai, China) with oligo (dT) 18 primers. Real-time polymerase chain reaction was performed in an ABI ViiA 7 Real-Time PCR System using the FastStart Universal SYBR Green Master (Roche, Shanghai, China). The amplification protocol consisted of one cycle at 95°C for 10 min, followed by 40 cycles of 95°C for 30 s, 60°C for 1 min, and 72°C for 1 min. A melting curve analysis was conducted post-amplification to ensure the specificity of the amplified product. The primers used in this study are listed in Table 1.

**TABLE 1:** RT-PCR primer sequence.

Gene	Primer sequence (5′-3′)	
Vimentin	Sense	CGT​CCA​CAC​GCA​CCT​ACA​G
	Antisense	GGG​GGA​TGA​GGA​ATA​GAG​GCT
MMP9	Sense	GCA​GAG​GCA​TAC​TTG​TAC​CG
	Antisense	TGA​TGT​TAT​GAT​GGT​CCC​ACT​TG
Acta2	Sense	CCC​AGA​CAT​CAG​GGA​GTA​ATG​G
	Antisense	TCT​ATC​GGA​TAC​TTC​AGC​GTC​A
Col1a1	Sense	CTG​TAA​CAT​GGA​AAC​TGG​GGA​AA
	Antisense	CCA​TAG​CTG​AAC​TGA​AAA​CCA​CC
β-actin	Sense	GGC​TGT​ATT​CCC​CTC​CAT​CG
	Antisense	CCA​GTT​GGT​AAC​AAT​GCC​ATG​T

#### 2.2.7 Western blot analysis

For protein analysis in renal tissues, samples, RIPA buffer (Beyotime, China) and protease inhibitor (PMSF, Biosharp, China) were homogenized and centrifuged at 14,000 rpm for 15 min at 4°C. Protein concentrations in the supernatant were measured using the Pierce BCA Protein Assay Kit (Thermo Fisher Scientific). After SDS-PAGE separation, 50 µg of protein was transferred onto a PVDF membrane. The membrane was blocked with 5% skim milk at room temperature for 1 h and then washed with TBST. Rabbit monoclonal antibodies against α-SMA (Proteintech Group, China; #14395-1-AP, 1:2000), TGFβ(ABclonal, China; #A23262, 1:2000), Col1a1 (ABclonal, China; #A24112, 1:1,000), and β-actin (ABclonal, China; #AC026, 1:500) were added and incubated overnight at 4°C. After another washing step, the appropriate secondary antibodies were applied, and the ECL kit (BIOPRIMACY, China, PMK0448) was used for visualization. The grey values of the protein bands were analyzed using the Gel Imaging System, and relative protein expression levels were normalized to β-actin as an internal control.

### 2.3 Statistical analysis statistical analysis

All data were statistically analyzed and visualized using GraphPad Prism 8.0 (GraphPad Software, Boston, Massachusetts, United States, www.graphpad.com). Data are expressed as mean ± standard deviation of the mean (SD) and were compared using one-way analysis of variance (ANOVA) followed by a *post hoc* Tukey test. Statistical significance was set at P < 0.05.

## 3 Results

### 3.1 Network pharmacology and reverse network pharmacology

#### 3.1.1 Identification of disease targets and shared targets

**FIGURE 2:**
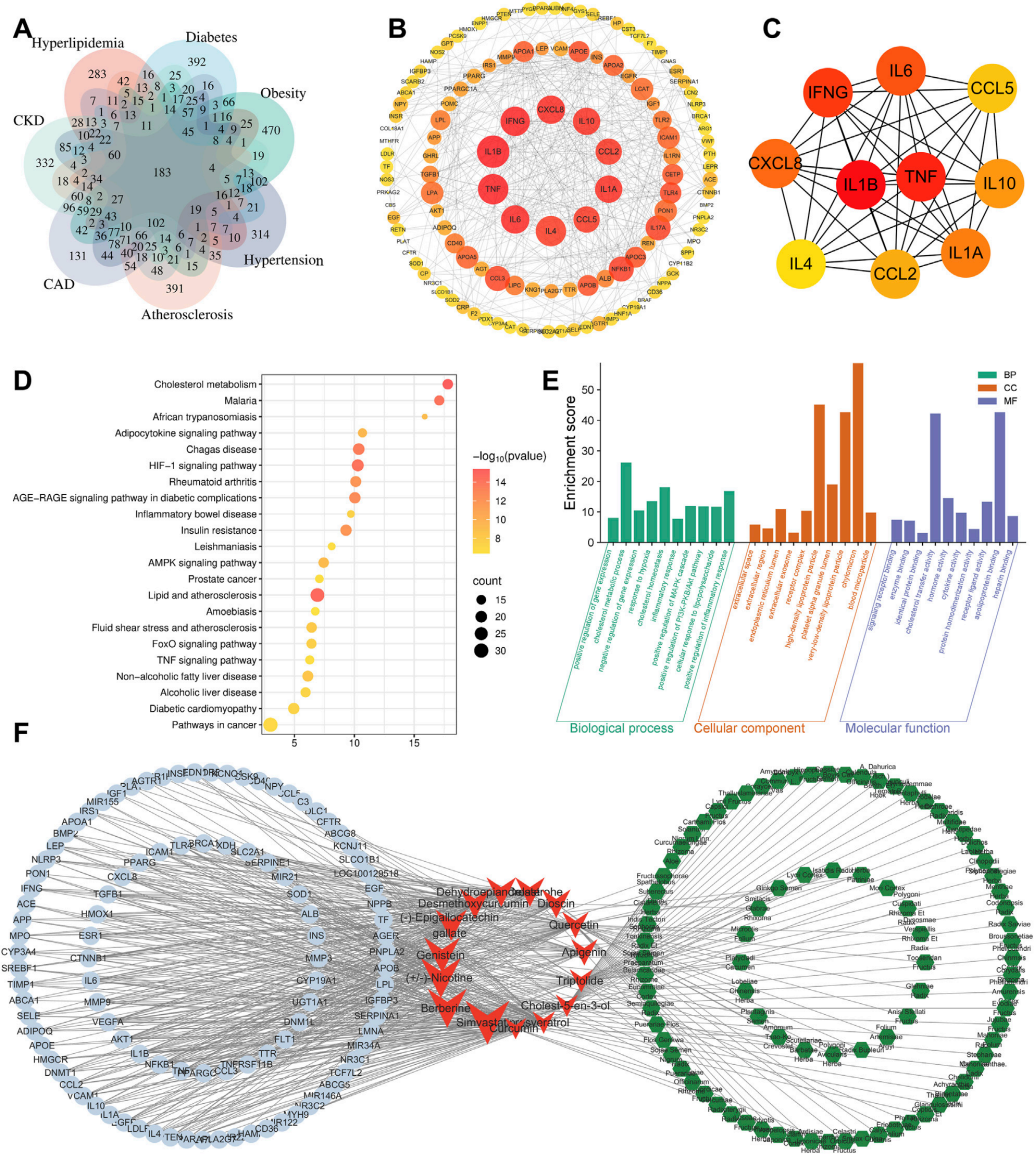
Bioinformatics analysis. **(A)** Intersection targets of CKD, CAD, HBP, and other CKM-related diseases. **(B)** PPI network diagram of intersection targets (tends to be red for high scores). **(C)** Core targets were screen out by Cytohubba plugin. **(D)** KEGG analysis. **(E)** GO analysis. **(F)** Target-compound-herb network (blue circles represent targets; red arrows represent compounds; green hexagons represent traditional Chinese herbs).

The number of targets obtained for “hyperlipidemia,” “diabetes,” “obesity,” “hypertension,” “atherosclerosis,” “coronary heart disease,” and “CKD” were 1,158, 1,565, 1719, 1856, 1,598, 1837, and 1930, respectively. Taking the intersection of these targets, we obtained 183 common core intersecting targets (Figure 2A); (Supplementary Table S1).

##### 3.1.1.1 PPI network construction

The results showed that the core targets of CKM were mainly IL-1β, TNF-α, IFN-γ, IL-6, CXCL8, IL-1A, IL-10, CCL2, CCL5 and IL4. These were considered as key targets (Figure 2C). The color intensity and size of each node represent their potential roles in the CKM context. Cluster analysis was performed on the PPI network using the Cytuhubba plug-in. The degree centrality and closeness centrality values indicated that a total of 42 core genes were identified (Figure 2B).

#### 3.1.2 KEGG and GO analysis

KEGG and GO analysis of 183 intersection targets and their corresponding genes for the 7 diseases were performed, as shown in (Figures 2D,E). The 183 core targets were imported into DAVID platform for GO analysis and KEGG analysis. Finally, 525BP (biological processes) related entries were obtained from GO analysis, and the top ten included: positive regulation of gene expression, cholesterol metabolic process, negative regulation of gene expression, response to hypoxia, cholesterol homeostasis, inflammatory response, positive regulation of MAPK cascade, positive regulation of phosphatidylinositol 3-kinase/protein kinase B signal transduction, cellular response to lipopolysaccharide, positive regulation of inflammatory response. There are 76 CC-related entries, and the top five includes: extracellular space, extracellular region, endoplasmic reticulum lumen, extracellular exosome, and receptor complex. There are 141 MF related entries, and the top five include: signaling receptor binding, enzyme binding, identical protein binding, cholesterol transfer activity, hormone activity.

There are 132 KEGG-related pathways. The top 10 include: Cholesterol metabolism, Lipid and atherosclerosis, HIF-1 signaling pathway, Malaria, Chagas disease, AGE-RAGE signaling pathway in diabetic complications, Insulin resistance, Rheumatoid arthritis, Adipocytokine signaling pathway, and AMPK signaling pathway.

#### 3.1.3 Reverse network pharmacology

The 183 targets were imported into the BATMAN-TCM database for analysis, which is a comprehensive database designed to predict the molecular mechanisms of traditional Chinese medicine (TCM) by integrating systems biology and pharmacology (Kong et al., 2024). This analysis aimed to obtain compounds that may cover the relevant targets, which were identified through the construction of a “target-compound-herb” network (Figure 2F). The top 5 compounds included: Simvastatin (Enrichment ratio = 33), BBR (Enrichment ratio = 29.98), 3-(1-Methylpyrrolidin-2-yl) pyridine (Enrichment ratio = 26.18), Genistein (Enrichment ratio = 23.04), and (−)-Epigallocatechin gallate (Enrichment ratio = 22.79). The top 5 herbal enrichments included Semen armeniacae amarum (KU XING REN), Smilax glabra (TU FU LING), Lycium chinense (GOU QI ZI), Panax ginseng (REN SHEN), and Ziziphus jujuba (DA ZAO), all of which contained 21 TCM ingredients.

After reviewing the literature, Simvastatin was not considered. This decision was based on its long research duration, clear target of action, and proven significant therapeutic effects on CKD, AS, coronary heart disease, and other conditions, as demonstrated in extensive clinical and basic studies (Al-Rasheed et al., 2018). Nicotine, known for its pronounced pathogenicity, was also eliminated. Meanwhile, BBR, a commonly used traditional Chinese medicine for treating diarrhea, has gained widespread attention due to its significant anti-inflammatory, lipid-lowering, and blood sugar-lowering effects (Chen et al., 2022; Shrivastava et al., 2023; Wang H et al., 2024). Previous studies have also found that BBR can improve cardiac diastolic function and renal pathological outcomes (Guo et al., 2021; Yang et al., 2023). However, there is currently no direct evidence of BBR’s improvement on CKM. This study hypothesizes that BBR may exert therapeutic effects on cardio-renal-metabolic syndrome by targeting core pathways, with specific mechanisms to be explored.

### 3.2 Establishment of Cardiovascular-kidney metabolic syndrome model

#### 3.2.1 HFD and L-NAME induce metabolic profile changes

**FIGURE 3:**
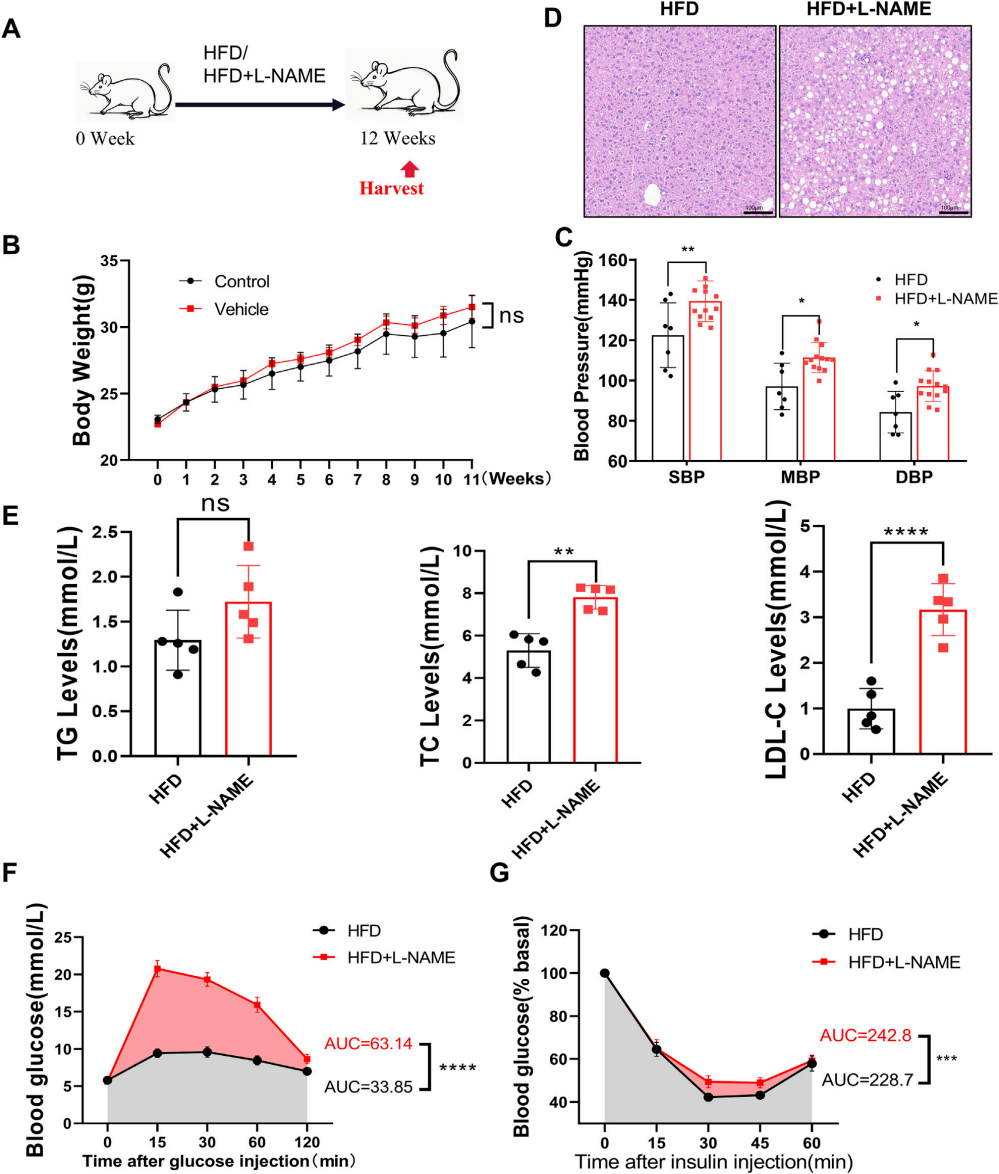
Construction of CKM mouse model: Metabolic and physiological function monitoring. **(A)** modeling process, C57 mice aged 12 weeks were fed HFD or HFD + L-NAME. **(B)** Body weight after treatment, no significant difference by T-test. **(C)** Systolic blood pressure at the end of modeling, comparison of mean and diastolic blood pressure. **(D)** Representative HE-stained liver images. **(E)** Quantitative analysis of lipid content, data expressed as mean ± standard deviation. **(F)** Area under the curve (AUC) for blood glucose changes and glucose tolerance test (GTT) between HFD and HFD + L-NAME groups. **(G)** Blood glucose changes and AUC for insulin tolerance test (ITT) between HFD and HFD + L-NAME groups. (mean ± SD, n = 5, P < 0.05 was statistically significant, ****P < 0.0001, ***P < 0.001, **P < 0.01 and *P < 0.05).

To investigate the metabolic impact of the HFD and L-NAME regimen, we monitored key physiological and biochemical parameters over a 12-week period. The HFD + L-NAME group exhibited a significant increase in systolic blood pressure, reaching levels that were notably higher than those observed in the control group (Figure 3C). Despite the elevated blood pressure, body weight remained relatively stable, with no significant differences between the groups (Figure 3B). Histological examination of liver sections revealed a marked accumulation of fat vacuoles in the HFD + L-NAME group, indicative of hepatic steatosis (Figure 3D). Furthermore, serum lipid profiles showed a significant rise in cholesterol levels, particularly in low-density lipoprotein cholesterol (LDL-C), while triglyceride levels remained within normal ranges (Figure 3E). Glucose tolerance tests (GTT) and insulin tolerance tests (ITT) further highlighted the metabolic disturbances, with the HFD + L-NAME group demonstrating reduced glucose tolerance and increased insulin resistance (Figures 3F,G). These findings collectively illustrate the profound metabolic alterations induced by the HFD and L-NAME regimen, setting the stage for the development of a comprehensive CKM phenotype.

#### 3.2.2 HFD and L-NAME lead to the development of a cardiovascular phenotype

**FIGURE 4:**
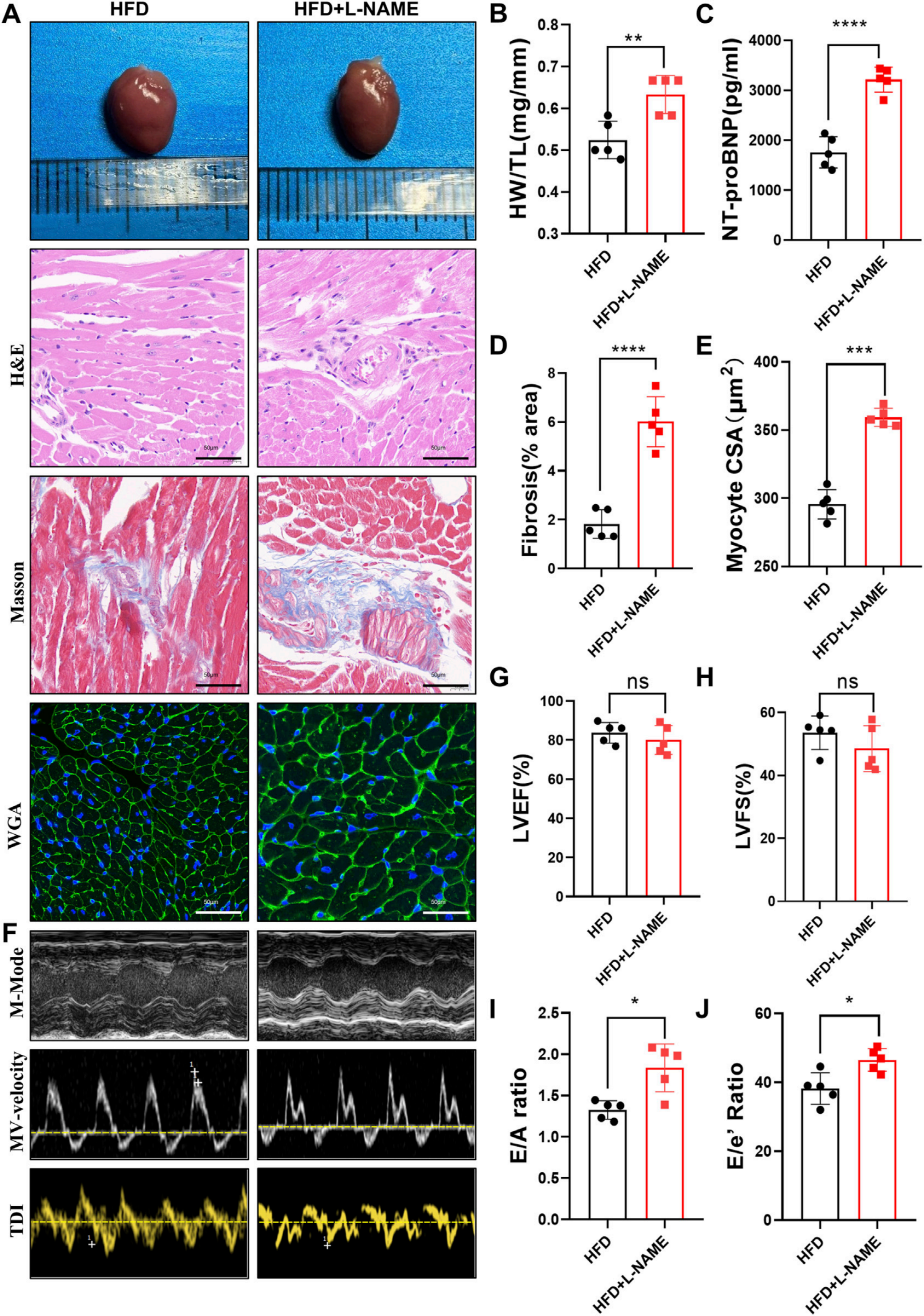
Construction of CKM mouse model:cardiovascular Part: **(A)** Typical morphological image comparison of, The representative images of GROSS(top), H&E, Masson and WGA(Bottom) staining of Heart. **(B)** The heart weight (HW) and tibial length (TL) were quantified, and the ratio of HW to TL was subsequently computed. **(C)**: serum NT-proBNP concentration **(D)** The proportion of collagen fiber area was calculated. **(E)** The cross sectional area (CSA) of cardiomyocyte analyzed by ImageJ software. **(F)** The representative LV M-mode echocardiographic tracings (top), pulsed-wave Doppler (Middle) and tissue Doppler (bottom) tracings. **(G)** Percent Left Ventricular ejection fraction (LVEF%). **(H)** Percent Left Ventricular Fractional Shortening (%). **(I)** Ratio of E/A. **(J)** Ratio of E/E’. (mean ± SD, n = 5, P < 0.05 was statistically significant, ****P < 0.0001, ***P < 0.001, **P < 0.01 and *P < 0.05).

The cardiovascular consequences of the HFD and L-NAME regimen were equally striking. Echocardiographic analysis revealed a significant increase in the heart-to-tibia length (HW/TL) ratio in the CKM mice, suggesting cardiac hypertrophy (Figures 4A,B). Elevated levels of NT-proBNP, a biomarker of cardiac stress, further corroborated the presence of cardiac dysfunction (Figure 4C). Masson’s trichrome histological staining highlighted extensive collagen deposition in the myocardium, indicating fibrosis (Figures 4A,D). Additionally, wheat germ agglutinin (WGA) staining revealed an increase in cardiomyocyte cross-sectional area, consistent with hypertrophy (Figures 4A,E). Functional assessment via echocardiography showed preserved systolic function, as evidenced by stable left ventricular ejection fraction (LVEF) and fractional shortening (LVFS), but a significant impairment in diastolic function, as indicated by reduced E/A and E/E’ ratios (Figures 4F–J). These results underscore the development of a distinct cardiovascular phenotype characterized by diastolic dysfunction and cardiac remodeling in the CKM mice.

#### 3.2.3 HFD and L-NAME result in structural damages and a decrease in kidney function

**FIGURE 5:**
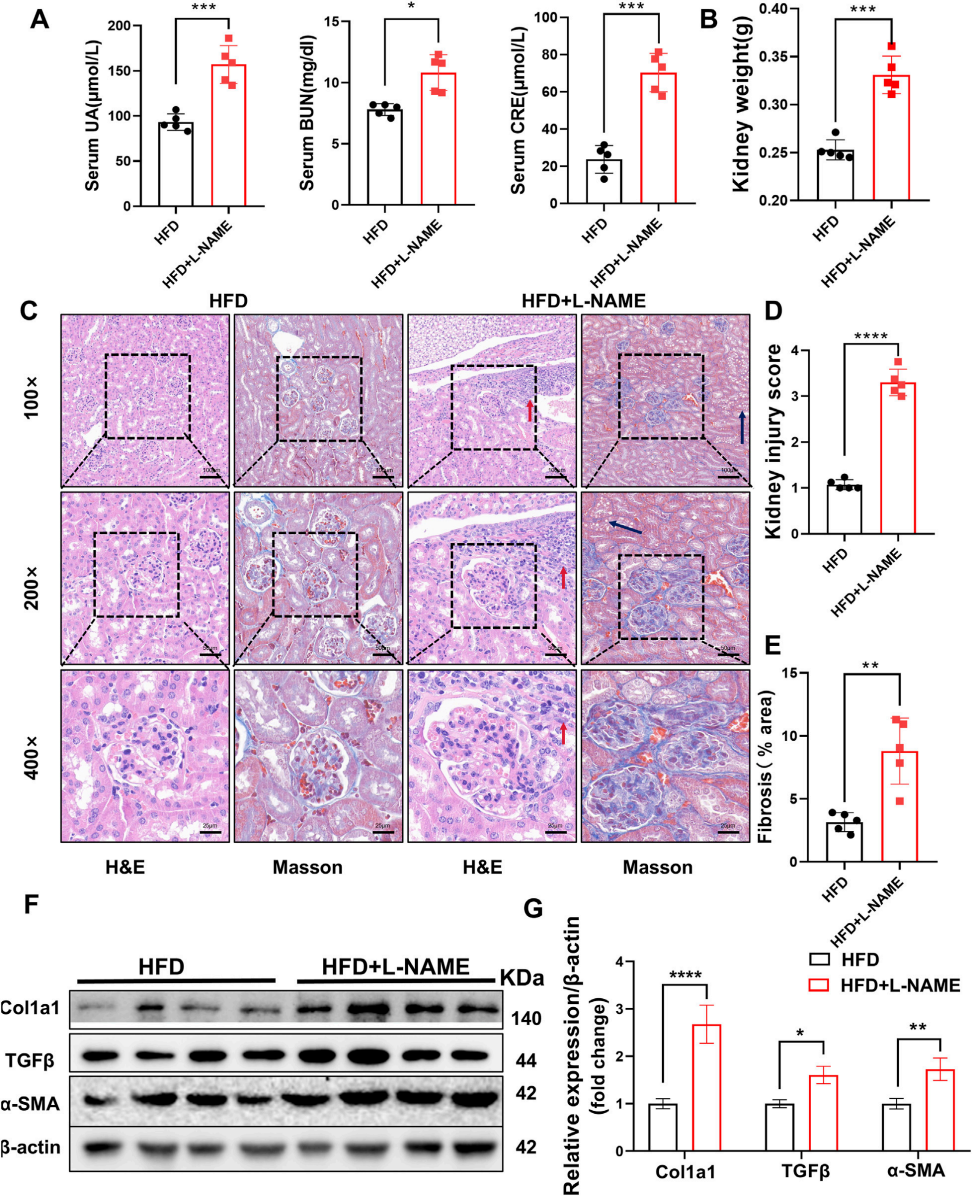
Construction of CKM mouse model: Renal Part: **(A)** Serum indicators of uric acid, creatinine, and urea nitrogen, **(B)** quantification of kidney weight, **(C)** typical images evaluated using two staining methods for Renal pathological changes: In the HFD group, glomeruli are normal in size and shape with clear boundaries. The glomerular capsule is very distinct. In the CKM group, glomeruli are deformed, enlarged, filled with blood cells, and capillaries are dilated. There is fat deposition in the renal tubular interstitium (black arrow), and inflammatory cell deposition (red arrow). MASSON staining shows significant fibrosis in the glomeruli and renal interstitium of the CKM group (blue area). **(D)** Renal injury score based on HE staining. **(E)**, semi-quantitative analysis of MASSON staining of the kidneys, including fibrosis area. **(F)**, Expression Analysis of fibrosis index:Representative Western blot images showing the protein expression levels of COL3a1, TGFβ, α-SMA and β-actin. **(G)**: Quantification of Western blot results. (mean ± SD, n = 5, P < 0.05 was statistically significant, ****P < 0.0001, ***P < 0.001, **P < 0.01 and *P < 0.05).

The renal impact of the HFD and L-NAME regimen was equally profound. Serum levels of creatinine, uric acid, and blood urea nitrogen (BUN) were significantly elevated in the CKM mice, indicating compromised renal function (Figure 5A). Gross examination revealed an increase in kidney weight, suggesting renal hypertrophy (Figure 5B). Histological analysis using hematoxylin and eosin (H&E) staining and Masson’s trichrome staining revealed extensive glomerular damage, interstitial fibrosis, and inflammatory cell infiltration (Figures 5C–E). Western blotting confirmed the upregulation of fibrosis-related proteins such as TGFβ, α-SMA, and Col1a1, further supporting the presence of renal fibrosis (Figures 5F,G). PCR analysis also revealed differences in the relative mRNA content of fibrosis-related genes (e.g., Acta2, Vim, Col1a1, and MMP9). HFD + L-NAME significantly increased the mRNA levels of these fibrosis indicators, but BBR improved these parameters (Figure 8H). These findings collectively highlight the significant structural and functional renal impairments induced by the HFD and L-NAME regimen, consistent with the development of a CKM phenotype.

### 3.3 The effect of BBR in CKM mice

**FIGURE 6:**
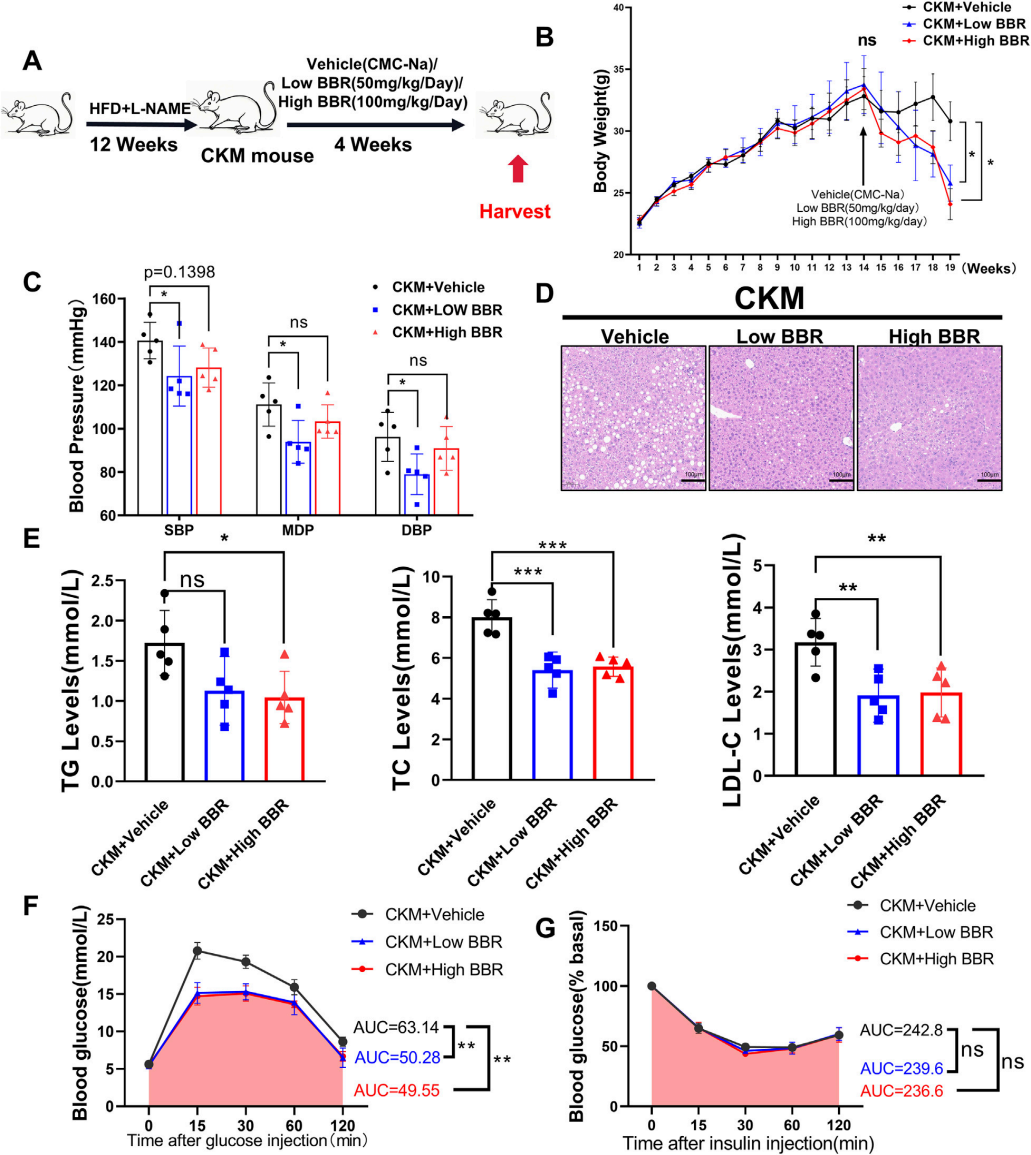
The effects of BBR on Metabolic and physiological function monitoring in CKM mice: **(A)** Experimental design **(B)** Body weight curve, after 4 weeks of administration, both high and low doses of BBR showed a decline compared to the media group with statistical differences (p = ??) **(C)** Representative HE staining images of the liver, showing a significant reduction in vacuoles formed by fat deposition compared to the control group. **(D)** Measured systolic blood pressure, diastolic blood pressure, and mean blood pressure in the BBR group were significantly decreased. **(E)** BBR significantly improved hyperlipidemia in CKM mice, including triglycerides (left), total cholesterol (center), and low-density lipoprotein (right). **(F)** Glucose tolerance test (GTT) changes and area under the curve (AUC) for the Vehile and BBR groups. **(G)** ns: No statistical significance. (mean ± SD, n = 5, P < 0.05 was statistically significant, ****P < 0.0001, ***P < 0.001, **P < 0.01 and *P < 0.05).

Using network pharmacology to screen BBR as a potential drug for CKM, we treated CKM mice with high and low doses for 4 weeks after modeling. We then observed and analyzed the relevant indicators to evaluate the therapeutic effects of BBR on CKM (Figure 6A).

#### 3.1 BBR exerts therapeutic effects on metabolic disorders in CKM mice

Berberine (BBR) emerged as a promising therapeutic agent in our study, demonstrating significant efficacy in mitigating the metabolic disturbances associated with CKM. BBR treatment led to a notable reduction in body weight, with both high- and low-dose regimens showing significant effects (Figure 6B). Notably, BBR significantly reduced systolic blood pressure in CKM mice, with the low-dose group showing a more pronounced effect compared to the high-dose group (Figure 6C). Histological examination of liver sections from BBR-treated mice revealed a marked reduction in hepatic fat accumulation, indicating improved lipid metabolism (Figure 6D). Serum lipid profiles further confirmed these findings, with significant reductions in triglycerides, total cholesterol, and LDL-C levels (Figure 6E). Additionally, BBR treatment improved glucose tolerance, as evidenced by increased area under the curve (AUC) during GTT (Figure 6F). However, BBR did not significantly improve insulin tolerance in CKM mice, as indicated by similar insulin tolerance test (ITT) results between the BBR-treated and control groups (Figure 6G). This suggests that while BBR has a significant impact on glucose tolerance, its effects on insulin sensitivity may be limited. These results highlight BBR’s multifaceted therapeutic effects on metabolic disorders in CKM mice.

#### 3.3.2 BBR improves cardiac structure and function in CKM mice

**FIGURE 7:**
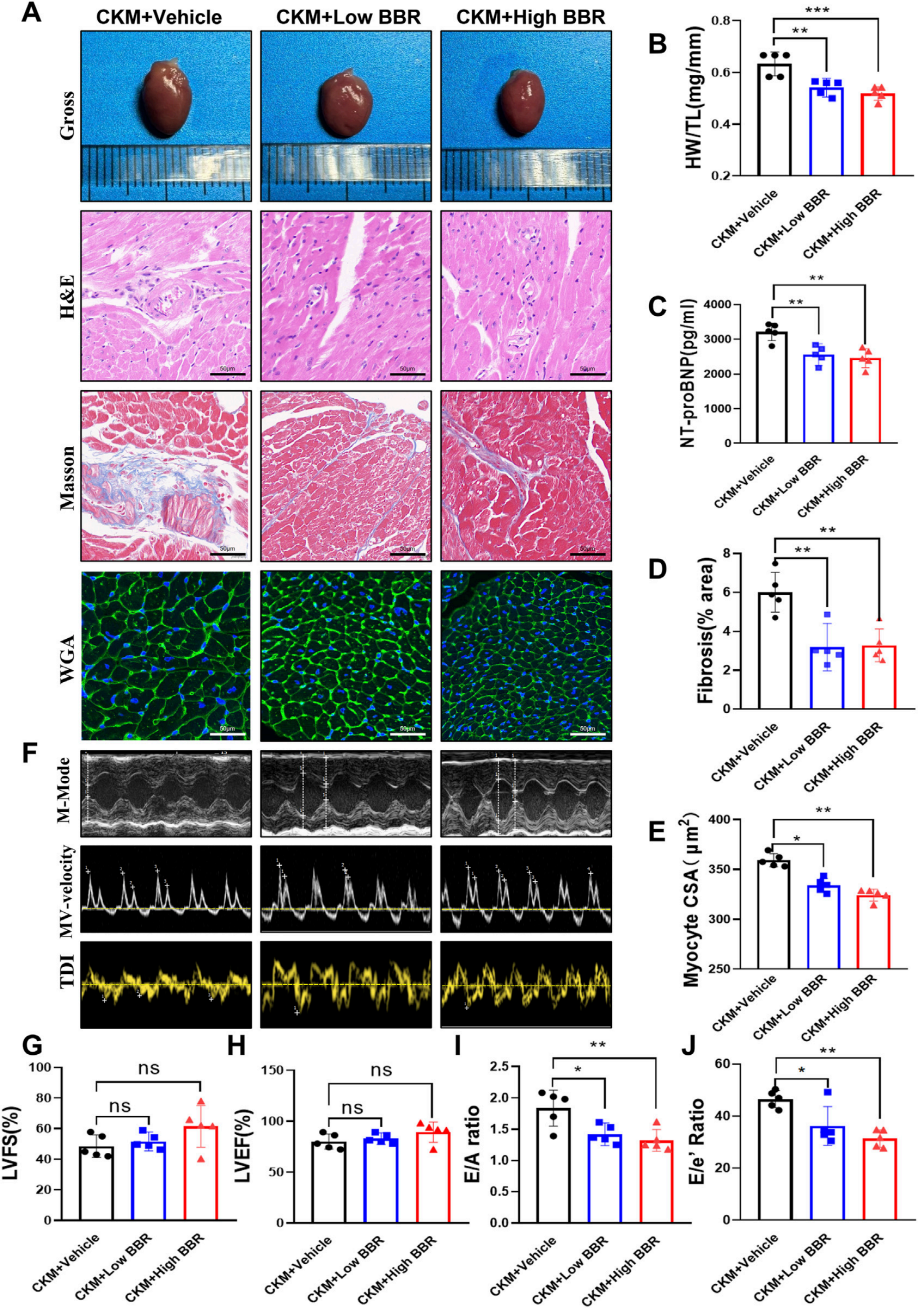
Effects of BBR on cardiac structure and function in CKM mice. **(A)** Representative gross views of the hearts in each group, along with representative patterns of H&E, Masson, and WGA staining. **(B)** Quantitative calculation of heart weight (HW) and tibia length (TL), and the ratio of HW to TL. **(C)** Representative images of heart staining. **(D)** Calculation of the proportion of collagen fiber area. **(E)** Analysis of myocardial cell cross-sectional area (CSA) using ImageJ Pro software. **(F)** Representative left ventricular M-mode echocardiographic tracking (top), representative pulsed Doppler (middle), and tissue Doppler (bottom) trajectories. **(G)** Left ventricular ejection fraction percentage (LVEF%). **(H)** Left ventricular shortening fraction percentage (LVFS%). **(I)** Ratio of E/A. **(J)** Ratio of E/E’. (mean ± SD, n = 5, P < 0.05 was statistically significant, ****P < 0.0001, ***P < 0.001, **P < 0.01 and *P < 0.05).

The therapeutic potential of BBR extended to the cardiovascular system, where it demonstrated significant benefits in improving cardiac structure and function. BBR treatment resulted in a reduced HW/TL ratio, indicating attenuation of cardiac hypertrophy (Figure 7B). Levels of NT-proBNP were significantly lower in BBR-treated mice, suggesting reduced cardiac stress (Figure 7C). Histological staining revealed a marked reduction in myocardial collagen deposition and improved cardiomyocyte cross-sectional area, consistent with reduced fibrosis and hypertrophy (Figures 7A,D,E). Echocardiographic analysis showed preserved systolic function, with stable LVEF and LVFS, but significant improvements in diastolic function, as indicated by increased E/A and E/E’ ratios (Figures 7F–J). These findings underscore BBR’s therapeutic effects on cardiac remodeling and function in CKM mice.

#### 3.3.3 BBR enhances renal function and reduces fibrosis in CKM mice

**FIGURE 8:**
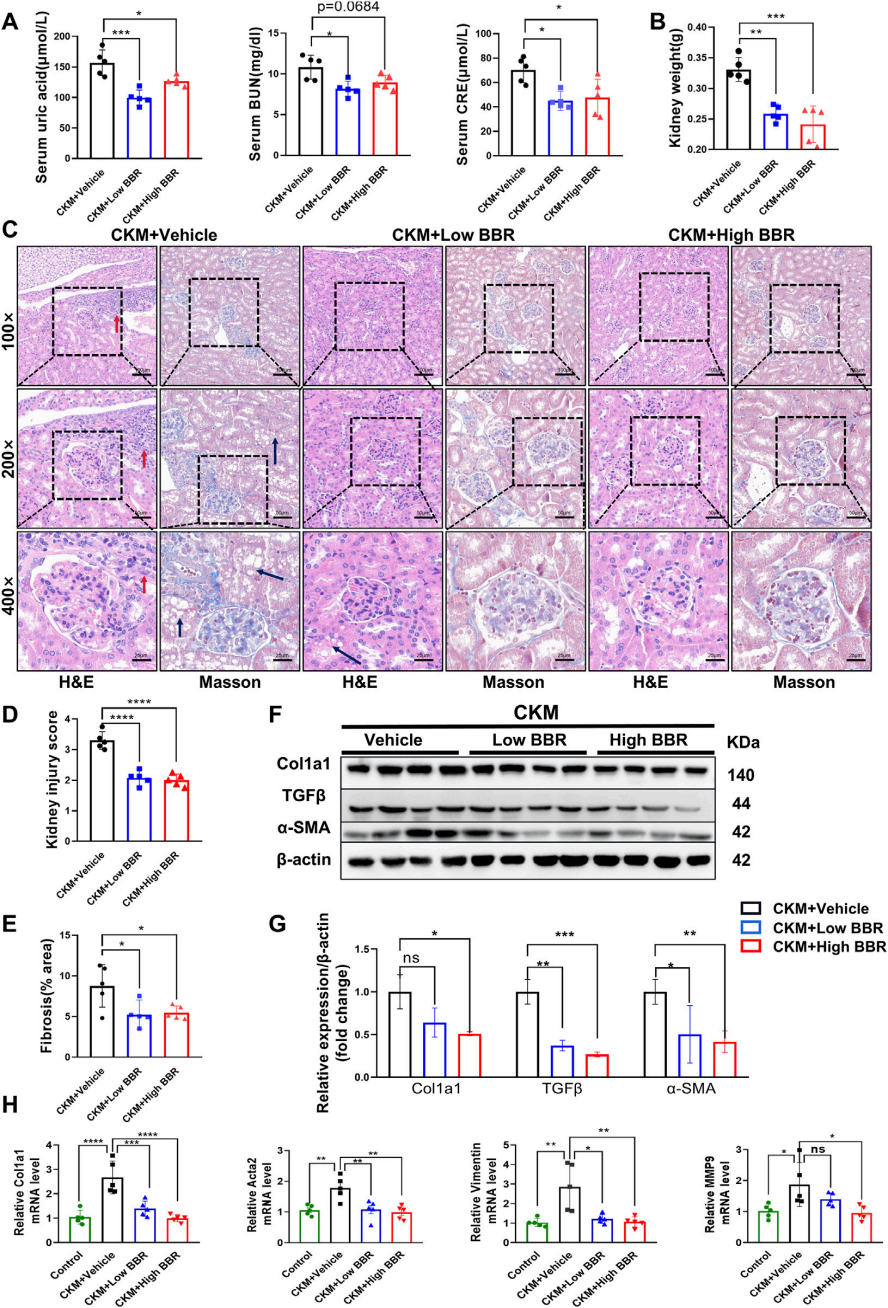
The effects of BBR on kidney function and fibrosis in CKM mice: **(A)** Serum indicators such as uric acid, creatinine, and blood urea nitrogen were significantly reduced in the low-dose BBR group compared to the high-dose group; **(B)** Typical images of Renal pathological changes evaluated using two staining methods: CKM + Vehicle shows glomerular deformation, increased volume, and engorged with blood cells, along with dilated glomerular capillaries. There is fat deposition in the renal tubular interstitium (black arrow), and inflammatory cell deposition (red arrow). MASSON staining reveals significant fibrosis in the glomeruli and renal interstitium of the CKM group (blue area). The CKM + BBR group shows better recovery compared to the CKM group; **(C)** Quantification of kidney weight; **(D)** Renal injury score based on HE staining. **(E)**, Semi-quantitative analysis of MASSON staining in the kidneys to determine the area of fibrosis. **(F)**, Expression Analysis of Relative mRNA expression levels of fibrosis markers:Representative Western blot images showing the protein expression levels of COL3a1, TGFβ, α-SMA and β-actin. **(G)**: Quantification of Western blot results. **(H)**: Vimentnt, Col1a1, MMP9, Acta2. (mean ± SD, n = 5, P < 0.05 was statistically significant, ****P < 0.0001, ***P < 0.001, **P < 0.01 and *P < 0.05).

The renal benefits of BBR were equally impressive, with significant improvements in both renal function and structure. BBR treatment led to a marked reduction in serum levels of creatinine, uric acid, and BUN, indicating enhanced renal function (Figure 8A). Gross examination revealed a reduction in kidney weight, suggesting attenuation of renal hypertrophy (Figure 8B). Histological analysis showed reduced glomerular damage, interstitial fibrosis, and inflammatory cell infiltration (Figures 8C–E). Western blotting confirmed the downregulation of fibrosis-related proteins such as TGFβ, α-SMA, and Col1a1, further supporting the therapeutic effects of BBR on renal fibrosis (Figures 8F,G). These results highlight BBR’s significant therapeutic effects on renal function and structure in CKM mice.

### 3.4 Validation of core targets and the effects of BBR

**FIGURE 9:**
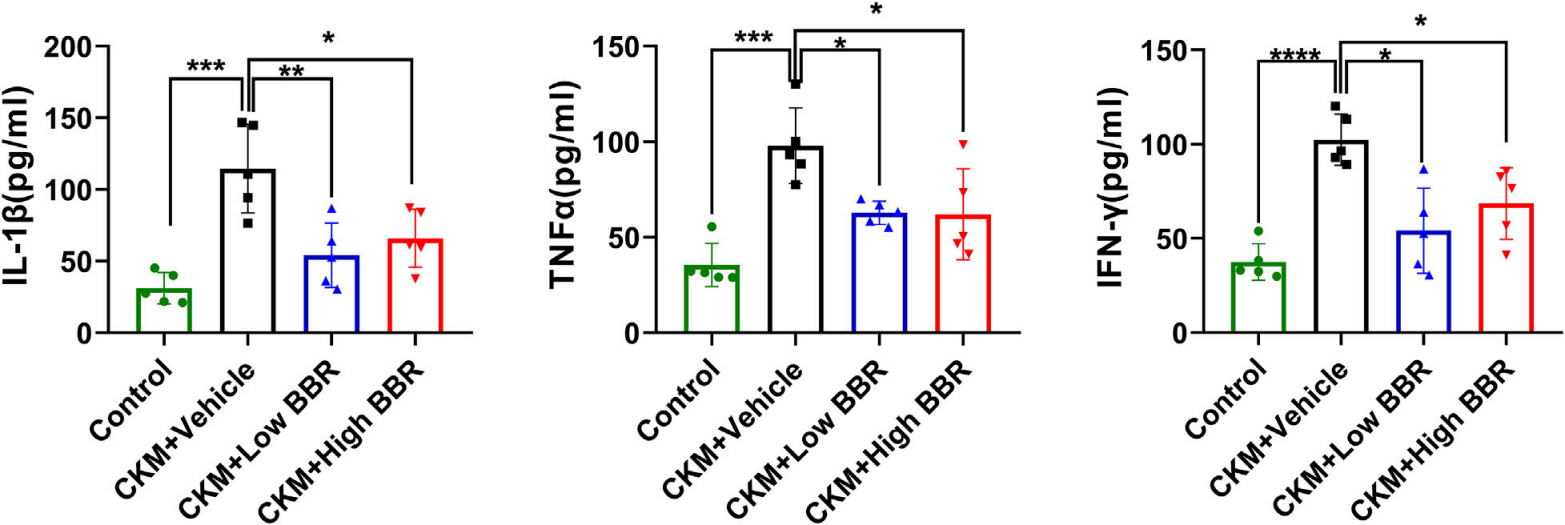
Validation of Core Targets and the Effects of BBR: After the establishment of the CKM model, the serum levels of IL-1β(left), TNF-α(middle), and IFN-γ(right) were significantly increased. However, both high and low doses of BBR significantly reversed this trend, with the low-dose group showing a more pronounced effect than the high-dose group. (mean ± SD, n = 5, P < 0.05 was statistically significant, ****P < 0.0001, ***P < 0.001, **P < 0.01 and *P < 0.05).

Serum levels of IL-1β, TNF-α, and IFN-γ were measured using the ELISA method. Compared with the Control group, the levels of these inflammatory cytokines were significantly elevated in the CKM + Vehicle group, indicating a significant increase in inflammation in the CKM model. BBR treatment significantly reduced the levels of these cytokines in both the low- and high-dose groups, demonstrating its anti-inflammatory effects in CKM mice (Figure 9).

## 4 Discussion

This study, starting from the pathophysiological mechanisms and disease progression of CKM, identified common targets through disease screening, followed by the selection of core targets and subsequent KEGG and GO enrichment analyses. The potential drugs were inferred from these common targets, and experiments were designed to validate them. In addition, this study systematically explored a novel “two-hit” mice model for CKM and then validated the therapeutic potential of BBR for CKM. The results showed that HFD + L-NAME can establish a stable CKM mice model. BBR significantly improved cardiac function, renal function, and fibrosis through multi-target effects. These findings provided new experimental evidence for the intervention of this complex syndrome.

Cardiovascular-Kidney Metabolic Syndrome (CKM) is a significant global health concern, associated with high morbidity and mortality rates and placing a substantial burden on healthcare systems (Savira et al., 2020; Kim et al., 2025). Over the past 20 years, the disease burden related to metabolic disorders has increased by 49.4%, making it a crucial risk factor for chronic kidney disease (CKD) and cardiovascular disease (CVD) (Xu et al., 2025). The treatment of CKM involves a multi-target, comprehensive management process, with coordinated interventions in cardiovascular, renal, and metabolic aspects. Several medications, such as sodium-glucose cotransporter 2 (SGLT-2) inhibitors and glucagon-like peptide-1 (GLP-1) receptor agonists, have been proven to improve CKM prognosis (Giugliano et al., 2022; Urbanek et al., 2023). However, poor patient adherence to multiple medications is a major barrier to effective treatment (Lim et al., 2023). Consequently, identifying effective therapeutic strategies to mitigate the adverse effects of CKM is of paramount importance. In this study, to explore the key targets and metabolites for CKM treatment, we integrated public databases and constructed a “target-compound-herb” network. This integration proved to be instrumental in pinpointing the crucial targets and metabolites linked to CKM.

The PPI network identified IL-1β, TNF-α, and IFN-γ as hub genes involved in the mechanism of CKM. IL-1β is a key driver of adipose tissue inflammation and systemic low-grade inflammation. In obese individuals, the expression of the IL1B gene in visceral adipose tissue is significantly elevated. It promotes the development of insulin resistance and metabolic syndrome by activating the NLRP3 inflammasome and NF-κB signaling pathways (Menini et al., 2020; Chen et al., 2023). IL1B also directly leads to myocardial fibrosis and tubulointerstitial damage in the kidneys by activating nuclear factor κB (NF-κB) and promoting the TGF-β signaling pathway. In chronic kidney disease (CKD) models, elevated IL1B levels are associated with glomerulosclerosis and proteinuria progression. Moreover, its positive correlation with angiotensin II (Ang II) further exacerbates cardiorenal crosstalk injury (Lei et al., 2019; de Alcantara Santos et al., 2021). Interestingly, although IL1B expression is significantly elevated in the visceral adipose tissue of obese patients, its levels are not directly correlated with diabetes status, suggesting that IL1B may independently initiate metabolic inflammation, separate from diabetes (Rakotoarivelo et al., 2018).

TNF-α is a classic pro-inflammatory cytokine secreted by adipose tissue, immune cells, and others, playing a central role in obesity, insulin resistance, and metabolic disorders (Sethi and Hotamisligil, 2021). TNF-α directly causes insulin resistance in skeletal muscle and adipose tissue by inhibiting the tyrosine phosphorylation of insulin receptor substrate 1 (IRS-1) and activating the IKK and JNK pathways (Bu et al., 2020). Additionally, TNF-α interacts with SGLT2 (sodium-glucose cotransporter 2), promoting vascular inflammation and endothelial dysfunction via the NADPH oxidase-angiotensin II pathway, thereby accelerating atherosclerosis (Mroueh et al., 2024). In cardiovascular diseases (such as myocardial infarction) and acute kidney injury (AKI), TNF-α exacerbates organ damage by promoting cardiomyocyte apoptosis and renal tubular epithelial cell necroptosis. Clinical data show that elevated TNF-α levels are significantly associated with mortality in patients with cardiorenal syndrome (Mavrakanas et al., 2017; Gergely et al., 2025). However, TNF-α inhibition may worsen cardiovascular risk in heart failure patients (Shanmugam et al., 2016; Rolski et al., 2023).

IFN-γ is a pro-inflammatory cytokine whose levels are elevated in patients with obesity and metabolic syndrome (MetS). For the kidneys, IFN-γ, in concert with APOL1 gene expression, can induce pyroptotic angiopathy in renal endothelial cells, accelerating the progression of chronic kidney disease (CKD) (Juliar et al., 2024). For the heart, activation of the IFN-γ signaling pathway in cardiomyocytes is associated with an age-related heart failure phenotype, characterized by metabolic reprogramming (such as inhibition of oxidative phosphorylation) and enhanced inflammatory responses (Ashour et al., 2023). In a nephrotoxic nephritis (NTN) model, anti-IFN-γ antibodies can reduce renal interstitial fibrosis (Kim et al., 2022).

KEGG enrichment analysis revealed that cholesterol metabolism, lipid and atherosclerosis, and the HIF-1 signaling pathway are closely associated with the development of CKM. Dysregulation of cholesterol metabolism plays a crucial role in atherosclerosis, metabolic inflammation, and renal dysfunction (Cortés-Camacho et al., 2024; Wang K et al., 2024). Cholesterol accumulation in renal tubular epithelial cells is closely related to glomerulosclerosis and tubulointerstitial fibrosis (Rampanelli et al., 2018; Yang et al., 2020). Abnormal renal lipid metabolism, such as cholesterol crystal deposition in renal tubules, can activate the renin-angiotensin system (RAS), promoting hypertension and renal fibrosis, thereby forming a vicious cardiorenal cycle (Agrawal et al., 2018; Handelsman et al., 2024). Inhibiting HMG-CoA reductase to lower LDL cholesterol can also reduce renal inflammation and oxidative stress (Park et al., 2017), and reducing remnant cholesterol can significantly improve the prognosis of CKM patients (Ding et al., 2025).

The HIF1α (hypoxia-inducible factor 1α) pathway has a complex pathophysiological link with CKM. HIF1α is a core regulator of cellular responses to hypoxia, but its activation in chronic metabolic diseases (such as diabetes and obesity) may exacerbate oxidative stress and mitochondrial dysfunction (Packer, 2020; Iacobini et al., 2022). In a mouse podocyte ablation model, HIF1α knockout can alleviate glomerulosclerosis and collagen deposition (Baumann et al., 2016). However, in chronic hypoxic or metabolically disordered states, persistent activation of HIF1α leads to fibrosis, inflammation, and organ dysfunction. For example, HIF1α-deficient vascular smooth muscle cells can significantly reduce vascular remodeling and macrophage infiltration (Qi et al., 2019; Xiao et al., 2024). In summary, HIF1α acts as both an adaptive response regulator and a pathogenic driver in CKM, with its effects highly dependent on the disease stage, tissue type, and metabolic environment.

Cardiovascular disorders, kidney disease, and diabetes are major health concerns that sharing common pathological mechanisms (Sebastian et al., 2024). Current research on CKM primarily focuses on clinical studies, examining specific drugs to improve the prognosis of the syndrome. However, fundamental investigations into its underlying mechanisms remain limited. In terms of basic research, CKM currently lacks a well-established model. Traditional HFD or diabetes models can only simulate metabolic-related diseases, with cardiac and renal involvement appearing later (Dragoljevic et al., 2021) The model induced by unilateral nephrectomy plus a high-sugar, high-fat, and high-salt diet for 12 weeks is the closest to the CKM phenotype (Carvalho et al., 2024). It can simulate chronic renal insufficiency combined with metabolic disorders, effectively mimicking the late-stage conditions of CKM. However, there are also drawbacks, such as high modeling costs and the inability to reflect early changes in metabolic syndrome of the heart and kidneys. Therefore, developing more economical and comprehensive CKM models remains an important direction for future research. This study is the first to utilize the classic heart failure with preserved ejection fraction (HFpEF) “two-hit” model to simulate CKM pathogenesis. It successfully reproduced renal function decline, insulin resistance, hypertension, cardiac diastolic dysfunction, and renal and cardiac fibrosis, providing new evidence for the CKM animal model.

In recent years, berberine has demonstrated significant potential in the treatment of cardiovascular diseases, kidney diseases, and metabolic-related disorders. Research in the field of cardiovascular diseases has shown that berberine possesses a range of therapeutic effects, including anti-heart failure, anti-arrhythmia, cholesterol reduction, inhibition of vascular smooth muscle proliferation, anti-platelet aggregation, and anti-inflammatory actions (Wang et al., 2015; Cookson, 2021; Abudureyimu et al., 2023; Hou et al., 2023; Song and Chen, 2021). For instance, in atherosclerosis models, berberine inhibits hepatic lipid synthesis enzymes (such as HMG-CoA reductase), promotes reverse cholesterol transport, and reduces serum total cholesterol (TC), low-density lipoprotein cholesterol (LDL-C), and oxidized low-density lipoprotein (ox-LDL). It also decreases aortic plaque formation and improves endothelial function (Tan et al., 2020). In ischemia-reperfusion models, berberine alleviates oxidative stress, autophagy, and inflammatory responses, thereby improving cardiac function after myocardial infarction (MI) and reducing cardiomyocyte apoptosis (Qing et al., 2018; Tian et al., 2023). Moreover, in the “two-hit” HFpEF model, berberine has been shown to mitigate myocardial diastolic dysfunction by modulating Drp1-mediated mitochondrial fission and Ca2+ homeostasis, which corroborates our findings (Abudureyimu et al., 2023).

Berberine has been shown to exert significant protective effects against kidney injury. It reduces serum creatinine and blood urea nitrogen levels, mitigates renal inflammation, and alleviates renal fibrosis (Yu et al., 2019). A meta-analysis revealed that berberine significantly improved renal function indicators (such as BUN and SCR) and inflammatory factors (IL-6 and TNF-α) in animal models of diabetic nephropathy (Hu et al., 2022). Additionally, berberine can lower blood glucose levels and reduce proteinuria (Yang et al., 2024). In chronic kidney disease (CKD) models, berberine modulates the gut microbiota by increasing the abundance of butyrate-producing bacteria and reducing the levels of uremic toxin-producing bacteria. It also inhibits the production of gut-derived uremic toxins, such as p-cresyl sulfate and trimethylamine N-oxide, thereby ameliorating the progression of chronic kidney disease (Pan et al., 2023).

Berberine plays a crucial role in regulating carbohydrate and lipid metabolism. Clinical studies have shown that berberine can reduce blood glucose levels and enhance systemic insulin sensitivity in patients with metabolic syndrome (Yu et al., 2019; Ilyas et al., 2020). In terms of lipid metabolism, berberine compounds (containing berberine, oryzanol, and vitamin B6) significantly lower serum triglycerides (TG) and LDL-C levels in rats with hyperlipidemia induced by a high-fat diet, and inhibit hepatic fat accumulation (Li et al., 2016). The mechanisms underlying these effects are primarily attributed to berberine’s activation of the AMPK pathway, which improves insulin resistance (IR) (Geng et al., 2016), and the AMPK/SIRT1 signaling pathway, which promotes adipose tissue browning and energy expenditure (Xu et al., 2021; Li et al., 2022, p. 3). Moreover, in diet-induced hyperglycemic mouse models, berberine enhances pancreatic β-cell function, significantly increasing insulin secretion and lowering blood glucose levels (Zhao et al., 2021). Notably, in our study, BBR significantly improved glucose tolerance but did not significantly enhance insulin tolerance, suggesting that its mechanism of action may be similar to that of acarbose, focusing more on inhibiting glucose absorption rather than improving insulin sensitivity (Carvalho et al., 2024).

As a crucial consideration for clinical use, the toxicity of berberine varies with species and administration route. For example, its intraperitoneal LD50 is 23 mg/kg, while the oral LD50 is 329 mg/kg (Singh and Sharma, 2018). At therapeutic doses, berberine shows low systemic toxicity with no severe organ damage or lethal reactions (Li et al., 2020; Sun et al., 2023). However, high doses or long-term use may cause liver and heart damage in zebrafish (Sun et al., 2023). Common adverse reactions include mild gastrointestinal symptoms like constipation and diarrhea (Imenshahidi and Hosseinzadeh, 2019). In a clinical study, 34.5% of T2DM patients treated with 500 mg of berberine three times daily for 13 weeks reported gastrointestinal issues, possibly due to its effects on gut microbiota (Yin et al., 2008). Berberine may also inhibit cytochrome P450 enzymes or P-glycoprotein, affecting drug metabolism, so caution is needed when co-administering certain drugs to avoid cumulative toxicity (Li et al., 2020).

Although this study has revealed the potential of BBR in the treatment of CKM, several limitations need to be addressed. Network pharmacology interprets diseases and drugs through known and predicted gene targets. However, it cannot provide specific genetic associations or causality with diseases as biological informatics methods like WGCNA or Mendelian randomization can. In this study, reverse network pharmacology enriched potential drugs, including nicotine, which is a factor causing the issue. Additionally, biological systems exhibit high complexity and nonlinearity. Network pharmacology, through network algorithm analysis, may not deeply involve core targets in disease progression; instead, it may only identify downstream common targets. This limitation may restrict the development of network pharmacology. These shortcomings are being addressed through multi-omics integration, such as metabolomics and transcriptomics combined validation (Huang et al., 2024). New methods, such as dynamic network modeling and AI-driven intelligent algorithm optimization (Zhang et al., 2024), have been gradually improved. However, they still lack systematic solutions.

Despite the fact that the HFD + L-NAME model partially mimics the pathological features of CKM, it may not fully capture the complexity and heterogeneity of human CKM. For instance, the model does not account for the interplay between genetic and environmental factors associated with CKM, which may limit the extrapolation of the study results. Meanwhile, although BBR has demonstrated promising therapeutic effects in animal experiments, the validation at the cellular and molecular levels is relatively insufficient. For example, there is a lack of direct evidence from genetic knockout or pharmacological interventions for key signaling pathways regulated by BBR (such as HIF1-α, AMPK and PI3K/AKT). This shortfall restricts the in-depth understanding of its mechanisms of action.

Future research directions include conducting clinical trials of BBR for CKM to evaluate its safety and pharmacokinetic characteristics in CKM patients. Mechanism expansion should involve using single-cell sequencing technology to decipher the regulatory networks of BBR on specific cell types in the heart and kidney, such as fibroblasts and podocytes. Additionally, exploring the synergistic effects of BBR with existing drugs, such as SGLT2 inhibitors, could optimize comprehensive management strategies for CKM.

## 5 Conclusion

This study provides comprehensive evidence that the HFD + L-NAME model effectively simulates the complex pathological features of Cardiovascular-Kidney Metabolic Syndrome (CKM), including metabolic disorders, cardiac dysfunction, and renal fibrosis. Through reverse pharmacology and multidimensional experiments, we identified berberine (BBR) as a potential therapeutic agent for CKM. BBR demonstrated significant therapeutic effects by improving metabolic profiles, enhancing cardiac diastolic function, and reducing renal fibrosis in CKM mice. These findings highlight BBR’s multi-target mechanisms, which simultaneously address multiple pathological aspects of CKM.

The results of this study underscore the potential of BBR as a novel therapeutic candidate for CKM, offering a new direction for the treatment of this complex syndrome. Future research should focus on further elucidating the molecular mechanisms underlying BBR’s therapeutic effects, particularly through single-cell sequencing and multi-omics approaches. Additionally, clinical trials are warranted to evaluate BBR’s safety and efficacy in human patients with CKM. These efforts will contribute to the development of more effective and targeted therapies for CKM, ultimately improving patient outcomes.

## Data Availability

All data generated in this study are included in the article/Supplementary Material. Additional inquiries can be directed to the corresponding authors.

## Abbreviations

Acta2, Actin, Alpha 2; ANOVA, Analysis of Variance; BBR, Berberine; CKD, Chronic Kidney Disease; CKM, Cardiovascular-Kidney Metabolic Syndrome; CMC-Na, Sodium Carboxymethyl Cellulose; Col1a1, Collagen Type I Alpha 1; E/A, Early Diastolic Velocity to Late Diastolic Velocity Ratio; E/E’, Early Diastolic Velocity to Tissue Doppler Ratio; GO, Gene Ontology; GTT, Glucose Tolerance Test; HFD, High-Fat Diet; HFPEF, Heart Failure with Preserved Ejection Frac tion; HW/TL, Heart Weight to Tibia Length Ratio; IFNγ, Gamma interferon; IL-1β, Interleukin-1β; ITT, Insulin Tolerance Test; KEGG, Kyoto Encyclopedia of Genes and Genomes; L-NAME, NG-Nitro-L-arginine methyl ester; LVEF, Left Ventricular Ejection Fraction; LVFS, Left Ventricular Fractional Shortening; MMP9, Matrix Metalloproteinase 9; NT-proBNP, N-Terminal Pro-B-Type Natriuretic Peptide; PPI, Protein-Protein Interaction; SD, Standard Deviation; SGLT2, Sodium-Glucose Cotransporter 2; TGFβ, Transforming Growth Factor Beta; TNFα, Tumor necrosis factor-α; Vimentin, Vimentin; α-SMA, Alpha-Smooth Muscle Actin.
